# Infrequent HPV Infection in Colorectal Neuroendocrine Carcinoma: Molecular and Histologic Characteristics

**DOI:** 10.3390/diagnostics15202569

**Published:** 2025-10-12

**Authors:** Xi Wang, Minghao Zhong, Xuchen Zhang, Yuanxin Liang

**Affiliations:** 1 Department of Pathology, Yale School of Medicine, New Haven, CT 06510, USA; 2 Department of Laboratory Medicine and Pathology, University of Minnesota, Minneapolis, MN 55455, USA

**Keywords:** colon, rectum, neuroendocrine carcinoma, human papillomavirus, histology, small cell, large cell

## Abstract

**Background/Objectives:** Colorectal neuroendocrine carcinomas (NECs) are rare, aggressive tumors with poorly defined clinicopathologic and molecular features. Their biological behavior and optimal treatment strategies remain unclear. Additionally, a subset of anorectal NECs may be associated with high-risk human papillomavirus (HPV) infection, suggesting potential heterogeneity in pathogenesis. **Methods:** We retrospectively reviewed 12 cases of colorectal NECs. Clinical outcomes, histologic morphology, immunohistochemistry, molecular profiling, including common oncogenic mutations, and HPV status were analyzed. **Results:** Seven cases demonstrated small cell NECs, and five showed large cell NECs. The majority of NECs (*n* = 9) arose in the rectum. *TP53* mutations were the most common (75%), followed by *KRAS, RB1, FBXW7*, and *BRAF* mutations. One case demonstrated mismatch repair (MMR) deficiency. High-risk HPV was detected in one rectal NEC, which lacked common oncogenic mutations and was the only long-term survivor (54 months). p16 expression did not correlate consistently with HPV in situ hybridization (ISH) status. Among small cell NECs with follow-up, platinum-based chemotherapy resulted in significantly longer survival than FOLFOX (13.5 vs. 4 months, *p* = 0.0209). **Conclusions:** Colorectal NECs display histologic and molecular heterogeneity. The tumors of small cell NECs potentially benefit more from platinum-based chemotherapy. HPV-associated NECs may represent a distinct subset with better prognosis, but p16 is not a reliable surrogate marker. Routine subclassification into small vs. large cell types and comprehensive molecular profiling, including HPV testing, may aid clinical decision-making and prognostication.

## 1. Introduction

Colorectal neuroendocrine carcinomas (NECs) are rare, comprising less than 1% of all colorectal cancers. Histologically, these tumors are characterized by poor differentiation and distinctive cytoarchitectural features, and they typically express immunohistochemical markers of neuroendocrine differentiation [1]. Clinically, colorectal NECs are highly aggressive and associated with poor prognosis. They are frequently diagnosed at an advanced stage, with a disproportionately high rate of distant metastases—significantly higher than that observed in conventional colorectal adenocarcinomas, where only about 25% of cases present with distant spread at diagnosis [2,3,4].

Colorectal NECs can be subclassified into small cell and large cell types based on histologic morphology. These two subtypes differ significantly in their clinicopathologic characteristics [5,6], and emerging evidence suggests distinct responses to chemotherapy between small cell and large cell carcinomas, primarily based on the data from lung NECs [7,8]. However, due to the rarity of colorectal NECs, comparative studies evaluating these subtypes in colorectal NECs remain lacking.

Human papillomavirus (HPV) is a well-established etiologic agent in a range of mucosal epithelial cancers, most commonly seen in the cervix but also identified in other anatomic sites [9,10]. Persistent infection with high-risk HPV subtypes plays a critical role in the carcinogenesis of premalignant lesions in these tumors [11,12]. Recent studies have identified a subset of anorectal NECs with molecular evidence of HPV involvement, suggesting an HPV-driven oncogenic pathway in a subset of cases [13].

In contrast to pulmonary NECs, which have been extensively characterized both histologically and genetically [14], colorectal NECs remain poorly understood [15]. Previous report suggests that anorectal NECs may arise through either HPV-dependent or HPV-independent mechanisms, displaying heterogeneous immunophenotypic and molecular profiles [13]. To date, no comprehensive studies have evaluated the histologic, morphologic, molecular, and immunophenotypic features of colorectal NECs—particularly those arising outside the anorectal region—or their potential association with high-risk HPV infection. This study aims to address this gap by characterizing the histologic, molecular, and immunophenotypic profiles of colorectal NECs and assessing the potential role of high-risk HPV in their pathogenesis.

## 2. Methods

A total of 12 cases of colorectal neuroendocrine carcinoma in our institute were retrieved in the time interval from January 2016 to December 2023 and retrospectively reviewed. The inclusion criteria were (1) a confirmed diagnosis of pure colorectal neuroendocrine carcinoma (mixed adenoneuroendocrine carcinomas were excluded); (2) availability of molecular and immunohistochemical profiling data; and (3) accessible clinical information.

Histologic features were assessed alongside molecular and immunohistochemical profiles. HPV in situ hybridization (ISH) staining was performed on most cases to evaluate the potential association between HPV infection and neuroendocrine carcinoma. All the features were presented as qualitative variables, while age, survival time, Ki proliferative index, and mitotic count were presented as quantitative variables.

All statistical analyses were performed using GraphPad Prism (version 8.0.0, San Diego, CA, USA). Continuous variables were compared using independent *t*-tests for two-group comparisons. Categorical variables were analyzed using the chi-squared test or Fisher’s exact test, as appropriate. Survival time was compared using Mantel–Cox tests for two-group comparisons. A *p*-value of <0.05 was considered statistically significant.

## 3. Results

### 3.1. Clinical Characteristics

Among the 12 patients in the study cohort, 2 were female and 10 were male. The mean age at diagnosis was 63.6 years. The majority of tumors were located in the rectum (*n* = 9), followed by the ascending colon (*n* = 2) and sigmoid colon (*n* = 1). At the time of initial diagnosis, 5 patients presented with distant metastatic disease. The median follow-up duration was 12 months (range: 2–42 months), and all patients developed distant metastases during disease progression.

Chemotherapy regimens primarily included either FOLFOX (*n* = 5) or platinum-based therapies (*n* = 6) (one patient lost follow-up). The median survival was 12 months in both treatment groups (*p* > 0.05). Notably, the only surviving patient at the last follow-up (54 months) was also the only case positive for high-risk HPV by ISH. Clinicopathologic features are summarized in Table 1.

### 3.2. Histologic Features of Colorectal Neuroendocrine Carcinoma

Based on morphologic criteria, 7 of the 12 cases displayed classic histologic features of small cell carcinoma, including cohesive round to oval cells with scant cytoplasm, finely granular chromatin (“salt and pepper” pattern), nuclear molding, and inconspicuous nucleoli, as defined by established diagnostic criteria [16]. Brisk mitotic activity, apoptosis, and necrosis are also noted in these tumors.

**Figure 1:**
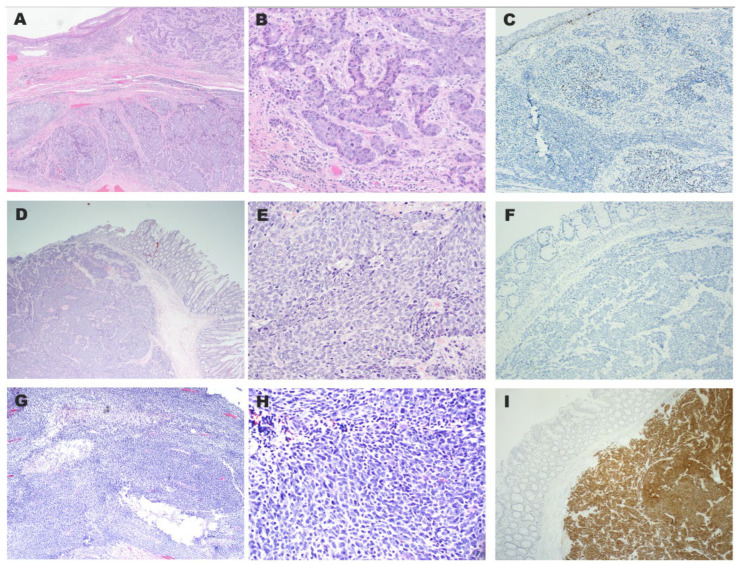
The HPV-positive neuroendocrine carcinoma showed large cell NEC morphology (**A**,**B**) and was positive for HPV high-risk ISH staining (**C**). One HPV-negative neuroendocrine carcinoma demonstrated large cell NEC morphology (**D**,**E**) and was negative for HPV high-risk ISH staining (**F**), while p16 immunostaining was positive (**I**). Another HPV-negative neuroendocrine carcinoma showed small cell NEC morphology (**G**,**H**).

Compared to large cell NECs, small cell NECs more frequently exhibited cytoplasmic absence (100% vs. 20%, *p* = 0.0038), hyperchromatic nuclei (100% vs. 20%, *p* = 0.0038), and molding (100% vs. 40%, *p* = 0.0180) (Figure 1). There were no statistically significant differences in the mutational profiles between small cell and large cell NECs (*p* > 0.05). Of the three cases with dual *TP53* and *RB1* mutations, two were small cell NECs.

There was no significant difference in chemotherapeutic regimens between the small cell and large cell NEC cases (*p* > 0.05). Treatment type also showed potential prognostic relevance. Among the six small cell NEC cases with available follow-up, those treated with platinum-based regimens had significantly longer survival than those treated with FOLFOX (13.5 months vs. 4 months, *p* = 0.0209). A summary of morphologic, immunophenotypic, and molecular features is presented in Table 2.

### 3.3. High-Risk HPV In Situ Hybridization and p16 Immunostaining

p16 immunohistochemistry is widely used as a surrogate marker for high-risk HPV infection [17,18]. High-risk HPV ISH was performed on most cases in our study cohort. The only HPV-positive tumor exhibited large cell NEC morphology and showed diffuse p16 expression. However, p16 immunohistochemistry did not consistently correlate with HPV ISH results. For instance, two HPV-ISH negative tumors also showed positive p16 staining, suggesting that p16 may be an unreliable surrogate marker for HPV status in colorectal NECs (Figure 1).

### 3.4. Genetic Alterations and Immunophenotypic Profiles

All tumors were positive for synaptophysin and at least one additional neuroendocrine marker, including chromogranin, CD56, or INSM1. p16 immunohistochemistry was performed in three cases, all of which were positive. The average Ki-67 proliferation index was 72%.

Genetic alterations were observed in the majority of cases. The most frequently mutated gene was *TP53* (*n* = 9), followed by *KRAS* (*n* = 4), *RB1* (*n* = 3), *FBXW7* (*n* = 3), *BRAF* V600E (*n* = 2), and *APC* (*n* = 1). Interestingly, the only HPV-positive case exhibited wild-type alleles for all the other genes tested.

Mismatch repair (MMR) deficiency was identified in one case, with concurrent loss of MSH2 and MSH6. PD-L1 expression (CPS < 1) was negative in most cases (*n* = 8), and all tested cases were HER2-negative. No statistically significant correlations were found between genetic alterations and known prognostic or therapeutic markers. Full molecular and immunohistochemical data are shown in Table 3.

## 4. Discussion

Neuroendocrine neoplasms (NENs) can arise in a wide range of anatomical sites and are defined by their expression of neuroendocrine markers. NENs encompass a spectrum from well-differentiated neuroendocrine tumors (NETs) to poorly differentiated neuroendocrine carcinomas (NECs), as well as pheochromocytomas and paragangliomas [19]. The most common primary sites include the gastrointestinal tract (62–67%) and the lungs (22–27%), but only less than 3% of NETs were classified as NECs [20,21]. Due to their rarity, histologic heterogeneity, and inconsistent nomenclature, the true incidence of NECs is difficult to ascertain [4]. Notably, up to 40% of NECs exhibit components of non-neuroendocrine histology; tumors in which the non-neuroendocrine component comprises more than 30% are classified as mixed adenoneuroendocrine carcinomas (MANECs) [22].

Colorectal NECs are aggressive tumors with poor prognoses. In our cohort, five of twelve patients had distant metastases at initial presentation, consistent with prior reports of advanced-stage disease at diagnosis [1]. While both rectum and ascending colon have been reported as common sites for NETs [23,24], most tumors in our series were rectal (*n* = 9), followed by the ascending colon (*n* = 2). This contrasts with some earlier studies that reported a predominance of NECs in the right colon [15]. The anatomic distribution in our study may reflect institutional referral patterns or geographic variation.

Histologically, NECs are conventionally categorized into small cell and large cell subtypes, paralleling the classification used for pulmonary NECs [6]. In our study, cases with small cell morphology showed classic features such as nuclear molding, hyperchromasia, and scant cytoplasm, consistent with the definition of pulmonary small cell carcinoma [16]. Colorectal small cell carcinoma (SmCC) is exceedingly rare, accounting for only 0.2–0.8% of colorectal malignancies [25,26,27]. Although extrapulmonary SmCCs are uncommon overall, they exhibit similar biological behavior and histologic features to their pulmonary counterparts [28,29,30].

Because small cell morphology is often treated with platinum-based regimens, we stratified NECs in our study into small cell and non-small cell groups. Response rates to platinum-based chemotherapy in extrapulmonary small cell carcinomas are reported at approximately 42%, lower than the 67% response rate seen in small cell lung cancer (SCLC) [4]. Some studies have suggested that 5-fluorouracil (5-FU)-based regimens such as FOLFOX may be effective for NECs [31], particularly in large cell NECs [32]. However, standardized treatment protocols for colorectal NECs remain undefined [15]. In our study, all patients received either FOLFOX or platinum-based chemotherapy. Although there was no significant difference in treatment regimens between morphologic subtypes overall, among patients with small cell features and available follow-up data, those treated with platinum-based regimens had significantly longer survival (13.5 months vs. 4 months, *p* = 0.0209). Even though the comparison of survival between treatment groups is limited by the small number of cases.

While our sample size was limited, genetic alterations showed some trends by histologic subtype. *TP53* mutations were more frequent among tumors with small cell features, whereas *KRAS* mutations were more commonly observed in non-small cell NECs. These findings are consistent with molecular profiles reported in pulmonary NECs. *TP53* mutations are found in up to 90% of SCLC tumors [33], while they occur in about 50% of large cell NECs [34]. In contrast, *KRAS* mutations are rare in SCLC (approximately 1.6%) [35]; however frequently found in colorectal adenocarcinomas (~40%) [36]. Furthermore, three cases in our cohort harbored dual *TP53* and *RB1* mutations, two of which demonstrated classic small cell morphology. This observation is consistent with prior studies showing *TP53/RB1* dual-mutations in 35–36% of pulmonary and extrapulmonary large cell NECs, while in more than 90% of SCLCs [37,38]. Interestingly, we also identified *KRAS* mutations (*n* = 2) and *BRAF* mutations (*n* = 2) in tumors lacking *RB1* mutations. Given that *KRAS* and *BRAF* are part of the RAS-RAF-MAPK signaling pathway frequently altered in colorectal adenocarcinoma [39], these findings support the hypothesis that NECs and adenocarcinoma components may be clonally related and originate from a common precursor cell [40].

Importantly, only three cases in our study were explicitly labeled as features of small cell carcinoma at diagnosis, except for the one without follow-up, all of which received platinum-based therapy [41]. This underlines a gap in pathology reporting that may impact treatment selection. We therefore recommend that pathologists include histologic subtype (small cell vs. large cell) when diagnosing colorectal NECs, as this distinction has potential therapeutic and prognostic implications.

Our study also highlights the presence of *FBXW7* mutations in three cases. Clinical studies have reported mixed responses to mTOR inhibitors in patients with *FBXW7*-mutated tumors [42]. Identifying this mutation in colorectal NECs may help guide potential targeted therapies and should be considered in molecular testing panels.

In addition, our findings support the concept of HPV-dependent and HPV-independent pathways in colorectal NECs. One rectal NEC case was positive for high-risk HPV by ISH and lacked mutations in *TP53, KRAS, APC, BRAF*, and *FBXW7*, suggesting a distinct molecular pathway of tumorigenesis. Previous studies have identified HPV as an etiologic agent in a subset of anorectal NECs [13], and our findings are in line with this model. Notably, the HPV-positive case in our study was the only patient alive at the last follow-up (54 months), raising the possibility that HPV-associated NECs may have a more favorable prognosis. Further investigation in larger cohorts is needed to validate this observation. p16 immunostaining, often used as a surrogate marker for high-risk HPV infection in other epithelial tumors [17,18], did not correlate well with HPV ISH results in our cohort. p16 overexpression can result from alternative mechanisms that dysregulate the Rb pathway, including genetic or epigenetic alterations affecting the *CDKN2A* gene (which encodes p16) or other genes involved in Rb signaling [43,44,45]. At least two HPV-negative cases were p16-positive, suggesting that p16 is not a reliable surrogate for HPV in colorectal NECs. Thus, direct testing via in situ hybridization or PCR-based methods remains essential for accurate HPV status determination in this setting.

## 5. Conclusions

In summary, our study characterizes the histologic, molecular, and immunophenotypic profiles of colorectal NECs and highlights the importance of subclassifying tumors into small cell and large cell types. We demonstrate the prognostic relevance of histologic subtype and suggest that platinum-based regimens may be more effective in small cell NECs. We also identify *FBXW7* mutations as potentially actionable targets. Finally, our data support the presence of an HPV-dependent subset of colorectal NECs, which may be associated with distinct molecular features and better outcomes. P16 is not a reliable surrogate marker for HPV in these tumors. Larger studies are needed to confirm these findings and guide future therapeutic strategies.

## Figures and Tables

**Table 1:** Clinicopathologic features of colorectal neuroendocrine carcinoma cases.

Case #	Tumor Site	Age	Gender	Histologic Features	HPV Ish	Stage at Diagnosis	Metastatic Sites During Progression	Primary Chemo Regimen	Follow-Up Months	Outcome
1	Ascending	76	Male	Small cell	Negative	M1 *	Multiple organs	FOLFOX	2	Died
2	Ascending	78	Male	Large cell	Negative	pT4a N2a	Liver	Platinum-based	11	Died
3	Sigmoid	48	Female	Small cell	Negative	pT4	Peritonium	Platinum-based	16	Died
4	Rectum	41	Male	Large cell	Negative	pT3N2b	Brain	FOLFOX	42	Died
5	Rectum	64	Male	Large cell	Negative	pT4	Multiple organs	FOLFOX	12	Died
6	Rectum	59	Male	Large cell	N/A	cT4bN2bM1	Bladder	Platinum-based	8	Died
7	Rectum	86	Male	Small cell	Negative	Lost follow-up				
8	Rectum	50	Male	Small cell	Negative	cT3N1M1	Liver	Platinum-based	14	Died
9	Rectum	59	Male	Small cell	N/A	cT4N1M1	Bone	FOLFOX	6	Died
10	Rectum	80	Female	Small cell	Negative	M1 *	Liver and Lung	Platinum-based	8	Died
11	Rectum	58	Male	Small cell	N/A	pT4	Multiple organs	Platinum-based	13	Died
12	Rectum	64	Male	Large cell	Positive	pT2N1b	Lung	FOLFOX	54	Alive

* The TNM stage is not available in the patient.

**Table 2:** Comparison of small cell and large cell NEC histologic features.

		Small Cell Neuroendocrine Carcinoma (*n* = 7)	Large Cell Neuroendocrine Carcinoma (*n* = 5)	*p* Value
Histologic features	Molding	100% (7/7)	40% (2/5)	0.0180
	Crush artifact	71% (5/7)	20% (1/5)	0.0790
	Tumor necrosis	71% (5/7)	60% (3/5)	0.6788
	Apoptosis	71% (5/7)	80% (4/5)	0.7353
	Cytoplasmic absence	100% (7/7)	20% (1/5)	0.0038
	Hyperchromatic nuclei	100% (7/7)	20% (1/5)	0.0038
	Nucleioli	14% (1/7)	57% (3/5)	0.0977
	Mitoses (per HPF)	7.3 ± 5.3	9.5 ± 6.6	0.5962
Immunostaining Profiles	Synoptophysin	100% (7/7)	100% (5/5)	1
	Chromogranin	43% (3/7)	40% (2/5)	0.9212
	Ki67 (%)	72 ± 27	72 ± 13	0.9695
Genetic Abnormalities	*TP53*	86% (6/7)	60% (3/5)	0.3105
	*Kras*	29% (2/7)	40% (2/5)	0.4140
	*APC*	14% (1/7)	0% (0/5)	0.3774
	*BRAF V600E*	29% (2/7)	0% (0/5)	0.1904
	*RB*	29% (2/7)	20% (1/5)	0.7353
	*FBXW7*	29% (2/7)	20% (1/5)	0.7353
Chemotherapy	Platinum based	67% (4/6) *	40% (2/5)	0.3765
Median survivle time (months)		10.5 (2–16)	12 (8–54)	0.1129

* The treatment information is not available in one patient who was lost to follow-up.

**Table 3:** Genetic abnormalities and immunoprofiles.

Case #	Histologic Features	HPV Ish	Synaptophysin	Chromogranin	Other Positive NE Markers	P16	Ki67	TP53	Kras	APC	BRAF V600E	RB	FBXW7	MMR	PD-L1 (CPS)	Her2
1	Small cell	−	+	−	CD56	N/A	30	Mutation	wildtype	wildtype	Mutation	wildtype	wildtype	Intact	<1	Negative
2	Large cell	−	+	−	CD56, INMS1	**+**	55	Mutation	wildtype	wildtype	wildtype	Mutation	wildtype	Intact	>10	N/A
3	Small cell	−	+	−	CD56	N/A	N/A	wildtype	wildtype	wildtype	Mutation	wildtype	wildtype	Intact	<1	N/A
4	Large cell	−	+	−	CD56, INMS1	N/A	60	Mutation	wildtype	wildtype	wildtype	wildtype	Mutation	Intact	>10	Negative
5	Large cell	−	+	−	CD56	N/A	81	wildtype	Mutation	wildtype	wildtype	wildtype	wildtype	Intact	>10	N/A
6	Large cell	N/A	+	+	CD56	N/A	90	Mutation	Mutation	wildtype	wildtype	wildtype	wildtype	Intact	<1	N/A
7	Small cell	−	+	−	INMS1	N/A	99	Mutation	wildtype	wildtype	wildtype	wildtype	wildtype	Intact	<1	N/A
8	Small cell	−	+	**+**	N/A	**+**	50	Mutation	wildtype	wildtype	wildtype	wildtype	wildtype	Intact	<1	Negative
9	Small cell	N/A	+	−	INMS1	N/A	N/A	Mutation	Mutation	wildtype	wildtype	Mutation	wildtype	MSH2 MSH6 loss	<1	N/A
10	Small cell	−	+	**+**	CD56, INMS1	N/A	99	Mutation	Mutation	wildtype	wildtype	Mutation	Mutation	Intact	<1	N/A
11	Small cell	N/A	+	**+**	N/A	N/A	80	Mutation	wildtype	Mutation	wildtype	wildtype	Mutation	Intact	<1	N/A
12	Large cell	**+**	+	**+**	INMS1	**+**	75	wildtype	wildtype	wildtype	wildtype	wildtype	wildtype	Intact	5–10	Negative

“+” = positive, “−” = negative.

## Data Availability

The raw data presented in this study are available on request from the corresponding author. The raw data are not publicly available becuase all the analyzed data has included in this article.

## Abbreviations

HPV: human papillomavirus; ISH: in situ hybridization; NEN: neuroendocrine neoplasm; NET: neuroendocrine tumors; NEC: neuroendocrine carcinomas; MANEC: mixed adenoneuroendocrine carcinoma; SmCC: small cell carcinoma; SCLC: small cell lung cancer; 5-FU: 5-fluorouracil
